# Development and Validation of an HPLC-UV Method for the Dissolution Studies of 3D-Printed Paracetamol Formulations in Milk-Containing Simulated Gastrointestinal Media

**DOI:** 10.3390/ph15060755

**Published:** 2022-06-16

**Authors:** Natalia Manousi, Christina Karavasili, Dimitrios G. Fatouros, Paraskevas D. Tzanavaras, Constantinos K. Zacharis

**Affiliations:** 1 Laboratory of Analytical Chemistry, School of Chemistry, Faculty of Sciences, Aristotle University of Thessaloniki, GR-54124 Thessaloniki, Greece; nmanousi@chem.auth.gr (N.M.); ptzanava@chem.auth.gr (P.D.T.); 2 Laboratory of Pharmaceutical Technology, Department of Pharmacy, Aristotle University of Thessaloniki, GR-54124 Thessaloniki, Greece; karavasc@pharm.auth.gr (C.K.); dfatouro@pharm.auth.gr (D.G.F.); 3 Laboratory of Pharmaceutical Analysis, Department of Pharmaceutical Technology, School of Pharmacy, Aristotle University of Thessaloniki, GR-54124 Thessaloniki, Greece

**Keywords:** paracetamol, HPLC, validation, accuracy profile, 3D printed, formulation, biorelevant media

## Abstract

Herein, a simple and rapid HPLC method for the determination of paracetamol milk-containing biorelevant media is proposed. The separation of the analyte from the milk-containing biorelevant media was accomplished isocratically using a mobile phase containing 25 mM phosphate buffer (pH = 3.0) and methanol, 80:20, v/v at a flow rate of 1 mL min^−1^. Following a protein precipitation-based sample clean-up, a thorough investigation of the effect of the precipitation reagent (methanol, acetonitrile, 10% v/v trifluoroacetic acid solution) on the analyte recovery was performed. The matrix effect was assessed in each biorelevant medium by comparing the slopes of the calibration curves of aqueous and matrix-matched calibration curves. The method was comprehensively validated using the accuracy profiles. The *β*-expectation tolerance intervals did not exceed the acceptance criteria of ±15%, meaning that 95% of future results will be included in the defined bias limits. The relative bias ranged between −4.5 and +3.9% for all analytes, while the RSD values for repeatability and intermediate precision were less than 2.7% and 3.0%, respectively. The achieved limit of detection (LOD) was 0.02 μg mL^−1^ and the lower limits of quantitation (LLOQ) were established as 10 μg mL^−1^, which corresponded to 2% of the highest expected concentration of paracetamol. The proposed scheme was utilized for the determination of paracetamol in dissolution studies of its 3D-printed formulation in milk-containing biorelevant media.

## 1. Introduction

The dissolution test is undoubtedly one of the most important studies during the process of new drug development. It is used to determine the kinetics of a drug’s release from its formulation and to provide information about its in vivo behavior [1]. This test is primarily used for quality control purposes and is typically performed in water or a buffer (with or without surfactants) as the dissolution medium according to Pharmacopoeia monographs [2]. Nevertheless, this “model” cannot always predict the in vivo behavior of the drugs, especially when the real dissolution medium is different (e.g., gastrointestinal environment). In order to simulate better the actual drug release in the physiological environment, “biorelevant” media can be prepared for providing a more realistic simulation of the fasted or fed gastrointestinal tract [3]. Popular biorelevant media—which are considered as benchmarks—involve the fasted-state simulated gastric fluid (FaSSGF), fasted-state simulated intestinal fluid (FaSSIF) and fed-state simulated intestinal fluid (FeSSIF). Mixing the former with full-fat milk as a key component (carbohydrates/proteins/fats) has proven to display similar physiochemical characteristics to the stomach after a meal [4].

Pharmaceutical technology based on 3D printing is an emerging area in the field of pediatrics. The customized pediatric-friendly medications with the desired shape and/or color improve the oral administration of drugs, which in turn could enhance their compliance [5]. Paracetamol (PAR) is a particularly common drug within the pediatric population and it is used to alleviate mild to moderate pain caused by headaches, toothache, etc., and for fever reduction as well [6]. In an effort to address the barriers to medication adherence among hospitalized pediatric patients, a child-friendly dosage form based on 3D-printed cereal was fabricated for the oral administration of paracetamol during breakfast [7]. Thus, it can be considered as an important biomarker in the fed state of in vivo–in vitro studies. Moreover, it is also useful for “biorelevant” in vitro dissolution tests, since these properties enable the study of the drug release kinetics of an ER tablet, overcoming any competing effects of drug partitioning into the fatty phase of the dissolution medium.

From an analytical chemistry point of view, developing reliable methods to support an in vitro dissolution study using biorelevant media is a challenging task [8]. Because of the matrix complexity, various sample preparation techniques have been published for quantifying various active pharmaceutical ingredients in complex media. Representative approaches involve liquid–liquid extraction (LLE) solid-phase extraction (SPE) [9], matrix solid-phase dispersion [10], solid-phase microextraction (SPME) [11] and ultrafiltration [12]. The majority of the aforementioned techniques are complex, time-consuming and typically implicate multiple steps. Protein precipitation [13] could be a simple and rapid alternative to the above and is based on the addition of a certain volume of a reagent (organic solvent, acid) in the milk-based biorelevant medium, leading to protein precipitation. A centrifugation and or filtration step usually follows prior to the analysis [14]. Due to the non-specific character of this sample cleanup scheme, selectivity and analysis efficiency are typically achieved by adopting a separation-driven analytical technique (HPLC, CE, GC, etc.) or analyte-specific mass spectrometric detection. To the best of our knowledge, there is only one report on the investigation of various drugs—including paracetamol—in fed-state gastric biorelevant media. This study focused on the theoretical correlation of the drug recovery with the physicochemical properties, and their report provided little information about analytical and validation data, such as precision, accuracy, etc. [15]. Consequently, there is a need for the development and validation of quantitative methods to determine paracetamol in samples obtained from a two-stage dissolution test of 3D-printed formulations in a complex medium.

The purpose of the present study was therefore to develop and validate a cost- and time-effective HPLC method for the quantitation of PAR in milk-based biorelevant dissolution media obtained from a 3D-printed innovative formulation intended for pediatric use. Due to the two-stage dissolution test, different biorelevant media were utilized, including simulated gastric and intestinal fluid. Following a protein precipitation-based sample cleanup, a thorough investigation of the effect of the precipitation reagent (methanol, acetonitrile, trifluoroacetic acid solution) on the analyte recovery was carried out. The HPLC method enabled the reproducible isolation of PAR from the complicated fat-rich medium and investigated analyte release during a two-stage biorelevant dissolution test. Method validation was implemented using the total error concept (to take into account the systematic and random errors) according to International Conference on Harmonization guidelines (ICH) [15].

## 2. Results and Discussion

### 2.1. Method Development

The establishment of efficient methods for the quantitation of PAR in milk-based biorelevant media was vital towards the development of novel techniques for drug delivery to children [7]. The USP monograph suggests the usage of UV batch spectrophotometry for the determination of PAR in dissolution samples. However, due to the matrix complexity of milk-based FaSSGF, FeSSIF and FaSSIF dissolution samples, the usage of separation techniques is mandatory for the reliable quantification of PAR. The USP-recommended HPLC-UV method for the assay is based on the usage of 0.1% acetic acid and methanol as mobile phases. In our case, we decided to replace the acidic modifier with a phosphate buffer (at the same pH value)—which offers higher capacity—in order to avoid any potential retention time-shifting and peak disturbances due to the analysis of samples with a wide range of pH values (pH 1.6 to 6.5 in gastric and intestinal simulated biorelevant media).

A systematic approach was followed to establish the appropriate chromatographic conditions for the fast and selective determination of PAR in certain samples. Selection of the LC column, mobile phase composition and pH value was investigated to obtain the optimum chromatographic conditions. Sufficient separation of PAR from the endogenous compounds of the sample matrix was achieved under isocratic elution using a 25 mM phosphate buffer adjusted to pH = 3 with H_3_PO_4_: CH_3_OH (80:20, v/v) using a flow rate of 1 mL min^−1^. Under the optimum experimental conditions, a symmetric PAR peak was achieved (T_f_ = 1.1), while the number of theoretical plates was N > 13,000. The runtime was set to 12 min. Extended analysis time up to 60 min evidenced no additional peaks, suggesting that PAR did not decompose in these media. Furthermore, the certain runtime was adequate for the elution of all matrix components. The retention time for PAR was 5.9 min.

### 2.2. Optimization of Sample Preparation

From a sample preparation point of view, the determination of drug content in milk-based biorelevant media is a challenging task as the components of the sample medium itself consist of lipids, proteins, carbohydrates, salts, etc., that have to be removed prior to HPLC analysis [14]. Analytical results may be influenced by the differential distribution of the drug in the multiple phases of a milk-based medium and it can also distribute in the aqueous or lipid phase or even bind to proteins/fats in the sample.

Among other sample preparation techniques utilized for extracting the analyte from milk-based samples, we adopted the protein precipitation approach as it is simple, easy and efficient [14]. However, one of the main drawbacks of this approach is its inability to completely remove the lipids of the particular matrices. Based on this matter, we kept the injection volume as low as possible (10 μL) and an extensive column wash step was followed at the end of each working day.

The performance of three different precipitation agents (MeOH, ACN, 10% v/v TFA) was studied on the recovery of the analyte in the examined matrices (FaSSGF: milk, FaSSIF: milk, FeSSIF: milk) at three different milk fat content levels, namely low fat (0.1%), medium fat (1.5%) and full fat (3.6%). The sample preparation protocol is described in Section 2.4. Each sample was spiked at five concentration levels of 10–600 μg mL^−1^ in order to assess the method’s efficiency in the entire determination range.

Assessing the potential matrix effect is of the utmost importance for the applicability of the procedure in milk-based samples. As characterized by IUPAC, the matrix effect is “the combined effect of all components of the sample other than the analyte on the measurement of the quantity”. This effect might be observed as an increase or decrease in the response compared with those obtained by aqueous solutions of the analyte, and it can be variable and unpredictable. To evaluate this parameter, a comparison of the slopes of the aqueous calibration curve versus the matrix-matched calibration curves prepared in each biorelevant media was conducted. The spiked samples in each biorelevant medium were prepared as the validation standards described in Section 2.3. As indicated in Table 1, the matrix effect was acceptable, varying between 90 and 110% in all cases, including the fat-rich medium (full-fat milk) and all different pH values of the biorelevant media (pH_FaSSGF_ = 1.6 and pH_FaSSIF/FeSSIF_ = 6.5) as well.

**Figure 1:**
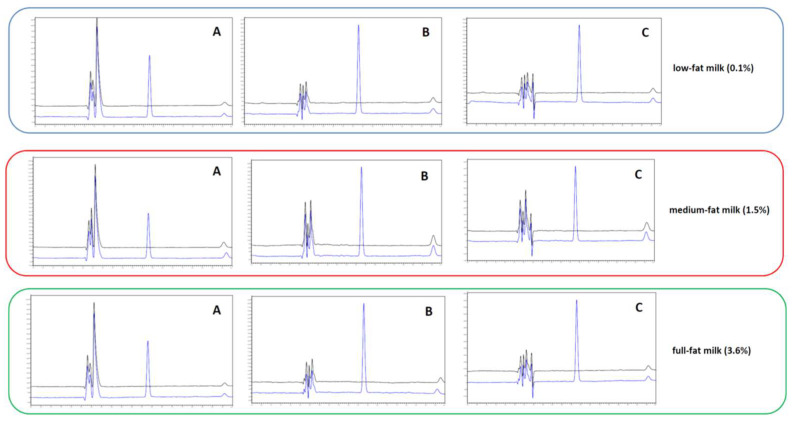
Representative overlaid chromatograms of blank FaSSGF:milk, 4:1, at different milk fat content levels (0.1, 1.5 and 3.6%) extracted with (**A**) 10% v/v aq. solution of TFA, (**B**) MeOH and (**C**) ACN. The chromatographic conditions are described in Section 2.2.

Since the observed variations (±10%) were entirely attributed to the experimental errors (day-to-day variation, etc.), there was no requirement to establish a correction factor (based on the drug recovery) for the determination of the dissolution profile. The results indicated that almost negligible protein/fat binding of PAR occurred. Analogous binding behavior of PAR has been also reported elsewhere [8,16]. The high aqueous solubility and low logP (0.30 [8]) mean that most PAR will be distributed to the aqueous phase. Based on the pK_a_ of PAR of 9.5, its molecule is expected to be fully protonated at the sample pH of both SGF and SIF. Figure 1 shows representative chromatograms of the blank FaSSGF: milk 4:1 medium at different milk fat content levels and spiked with (c = 10 μg mL^−1^).

Apart from the comparison of the calibration slopes, other experimental issues should be taken into consideration in the selection of the suitable precipitation reagent. When ACN and MeOH were utilized as protein precipitation reagents in both FaSSGF: milk and FaSSIF: milk mixtures, a “milky” solution was observed during the sample dilution step with water, causing their precipitation. This may be attributed to the decrease in the solvation of the residual fats and/or proteins when the organic solvent content reduces to ca. 10% in the final solution. To overcome this barrier, the mixture was then subjected to centrifugation for 15 min at 6000 rpm and filtrated to remove the denatured milk proteins and unwanted solids. Based on these observations, the 10% v/v TFA solution was finally chosen as the precipitation reagent to keep the entire procedure as simple as possible.

Looking at the chromatographic parameters, the number of theoretical plates of the PAR peak was—as expected—almost 20% higher in the case of TFA compared to the organic solvents (ACN, MeOH). Generally, peak fronting may occur due to the injection of a higher eluotropic strength of sample compared to the mobile phase [14]; however, in our case, the tailing factor remained practically unaffected.

### 2.3. Method Validation

The proposed method was validated to prove its suitability for its intended use, satisfying the anticipations described in the ICH Q2(R1) guideline [15]. The proposed HPLC protocol was validated using accuracy profiles based on the SFSTP Commission proposal [17].

#### 2.3.1. System Suitability

The system suitability was investigated with six replicate analyses of 10 μg mL^−1^ PAR solution. The %RSD values for the peak area and retention time were 1.2 and 0.8%, respectively, which are within the acceptance criterion of ±2%. Under the optimum conditions, the peak tailing was found to be less than 1.1 and the number of theoretical plates was more than 5000 (ca. 13000), indicating the acceptability of the peak characteristics.

#### 2.3.2. Selectivity and Carry-Over

**Figure 2:**
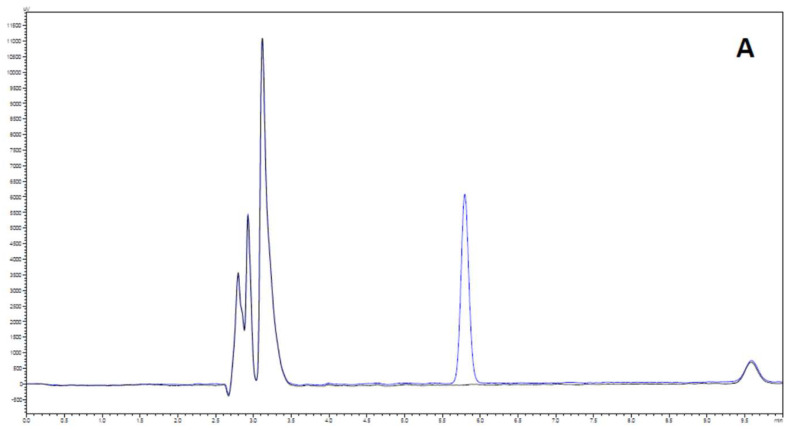

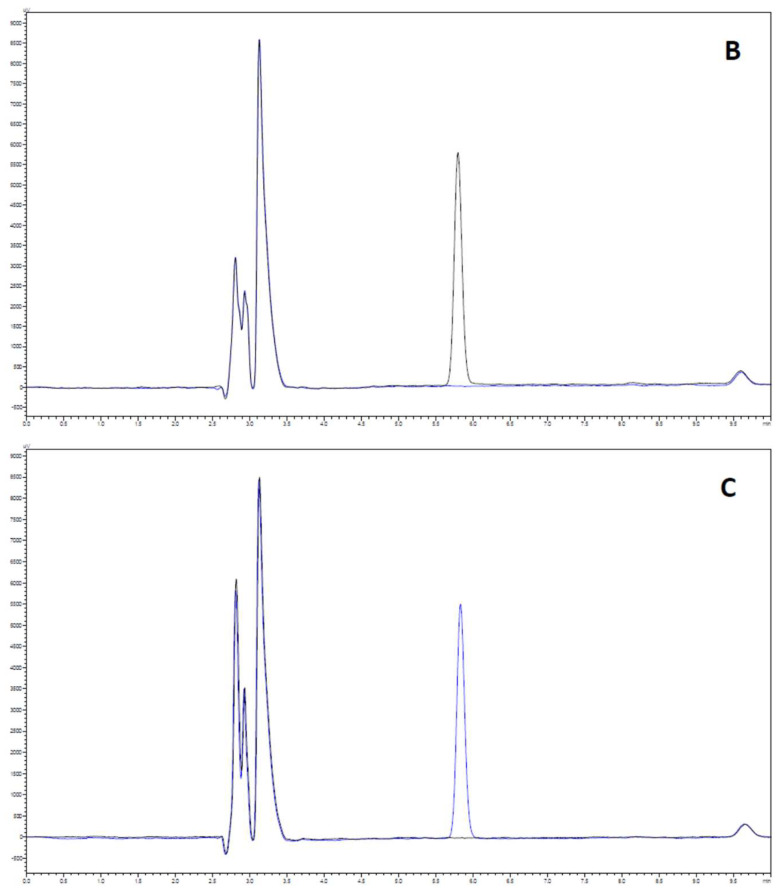
Representative HPLC chromatograms of the analysis of blank and spiked samples (with 10 μg mL^−1^ PAR). (**A**) FaSSGF: milk (4:1); (**B**) FaSSIF: milk (4:1); (**C**) FeSSIF: milk (4:1). Full-fat (3.6%) milk was used in all cases.

Method selectivity was assessed through the analysis of blank and spiked samples (FaSSGF: milk, FaSSIF: milk, FeSSIF: milk, 4:1 in all cases) with PAR (c = 10 μg mL^−1^). The HPLC method showed good chromatographic separation of PAR and milk-based biorelevant media. No interferences were observed in the tested matrices. As depicted in Figure 2, PAR was eluted as a single peak and good resolution between PAR and the other peaks of the sample matrix was observed.

The potential carry-over effect was studied through the subsequent injection of spiked samples at the highest calibration level of 600 μg mL^−1^. No “ghost peaks” were recorded and no effect on the response of PAR was observed, suggesting that the washing procedure that was followed between the injections was sufficient.

#### 2.3.3. Selection of the Response Function

To prove that the proposed method is suitable for its intended use, the validation was performed using the total measurement error (systematic and random error) approach [18]. This concept includes the selection of the most appropriate calibration model. In order to find out the appropriate response model, the fitting of different regression models to the calibration standards was investigated. The mean relative bias, the intermediate precision (s_r_, %) and the upper and the lower *β*-ETI were calculated by using the back-calculated concentrations of the validation standards through each regression model. The suitability of these models was evaluated by plotting the accuracy profiles at a probability of 95%, while the acceptance limits of λ at ±15% level were considered [19].

**Figure 3:**
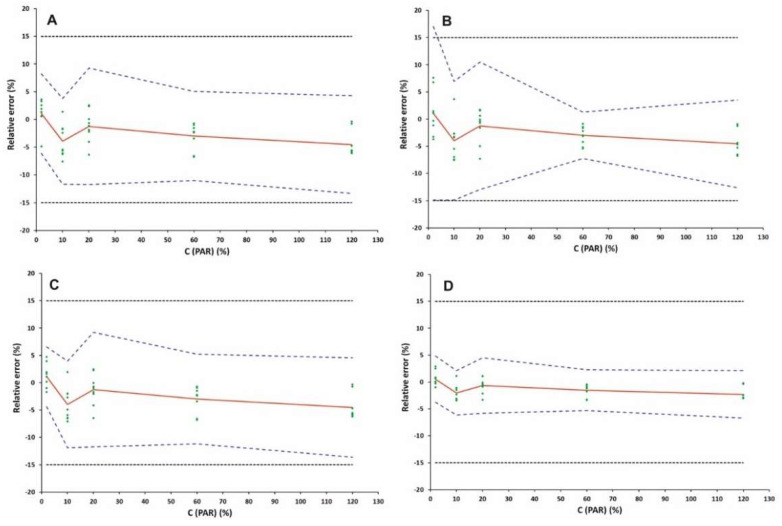
Accuracy profile for the determination of PAR using (**A**) linear unweighted regression model; (**B**) linear regression fitted only at the highest concentration (120% level); (**C**) weighted (1/X) linear regression; (**D**) linear regression after square root transformation. The relative error (%), the accuracy profile and the acceptance limits λ (±15%) are described by the red plain, blue dashed and blank dotted lines, respectively. The dotted curves show the acceptance limits λ ± 15%, the plain blank line shows the Y = X identity line, and the blue dashed line shows the accuracy profile (β-ΕΤΙ).

In our study, four different response functions comprising simple unweighted linear regression, linear regression fitted only at the highest concentration (120% level), weighted (1/X) linear and linear regression after square root transformation were investigated. In the case of linear regression forced through the origin using the 120% level (Figure 3B), the scattering of the data resulted in a spreading of the β-interval that might be higher or overpass the acceptance limit of 15% at the lowest concentration level of 2%. By using unweighted or weighted (1/X) linear regression and square-root-transformed regression (Figure 3A,C,D), the tolerance intervals were totally within the acceptance limits for the studied calibration levels.

For simplicity reasons, we finally selected the unweighted linear model, which provides the least bias and least extrapolation of the experimental results. The validation results are tabulated in Table 2.

#### 2.3.4. Linearity

**Figure 4:**
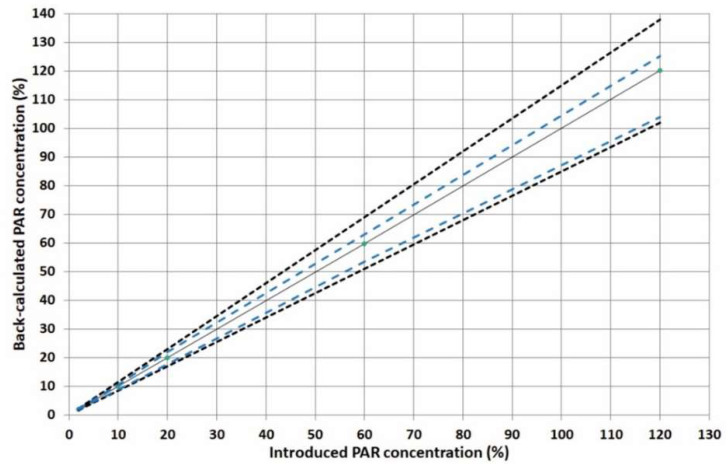
Linearity profile for PAR. The identity line (Y = X) corresponds to the plain blank line, the accuracy profile (β-ΕΤΙ) corresponds to the blue dashed line, and the acceptance limits λ ±15% correspond to the dotted curves.

The linearity was examined by plotting the back-calculated concentrations and introduced concentrations using a linear unweighted regression model in the specified calibration range. As shown in Figure 4, the tolerance interval was entirely within the acceptance limits for the examined calibration levels, providing sufficient performance regarding method precision and trueness even at the lowest calibration level of 2% (corresponding to 10 μg mL^−1^).

Method linearity is shown graphically in Figure 3. The slope was found to be close to 1, while the intercept was found to be close to zero. Moreover, the R^2^ value was found to be 0.9999, indicating the acceptable linearity of the developed analytical method.

#### 2.3.5. Accuracy, Precision and Trueness

The trueness of an analytical method is defined as the closeness of an experimental value derived from a set of replicated measurements and the conventionally accepted value [17], and it is typically expressed as relative bias (%). In this case, the relative biases were less than 15% (−4.5 to +3.9%) for all concentration levels, showing good method trueness.

The precision of an analytical method is defined as the dispersion of the replicated determinations, and it is described in terms of intermediate precision and in terms of repeatability (intra-day). Precision is expressed as the RSD of time-dependent intermediate precision (s_R_, %) and of repeatability (s_r_, %). As shown in Table 2, the s_r_ and s_R_ values were less than 2.7 and 3.1%, respectively, showing the good precision of the proposed method.

The accuracy of an analytical method is defined as the closeness of agreement between a conventional true or accepted reference value and the test results [15]. The accuracy considers the total error, which takes into consideration both the random and the systematic errors. In this case, the lower and upper *β*-ETIs were inside the acceptance limits of ±15% (which correspond to a probability level of 95%) for all concentration levels, showing that the utilization of the proposed protocol can lead to accurate results within the specified concentration range.

#### 2.3.6. LODs and LOQs

The limit of detection (LOD) was calculated as LOD = LLOQ/3.3 and it corresponds to the lowest amount of analyte that can be detected without being accurately quantified. The lower limit of quantification (LLOQ) and the upper limit of quantification (ULOQ) were obtained from the accuracy profiles. LLOQ reflects the minimum concentration at which *β*-expectation intervals fall outside the acceptance limits, and ULOQ reflects the maximum concentration at which *β*-expectation intervals fall outside the acceptance limits. In this case, the β-expectation intervals fall within the acceptance limit, and, as a result, the LLOQ and ULOQ were 2 and 120% (corresponding to 10–600 μg mL^−1^), respectively. The LOD was estimated to be 0.02 μg mL^−1^ based on the S/N = 3 criterion.

#### 2.3.7. Solution Stability

Proteins and fats typically have binding tendencies that may either make the drug unavailable for analysis or may affect the stability of the drug [14]. Based on this, the stability of PAR was examined in each sample medium (FaSSGF: milk, FaSSIF: milk, FeSSIF: milk) for 8 h at ambient temperature (25 °C). The results were calculated based on the % recovery against freshly prepared solutions. The % recovery was higher than 96.3%, concluding that the analyte was stable during the studied period.

### 2.4. Method Application—Dissolution Study

**Figure 5:**
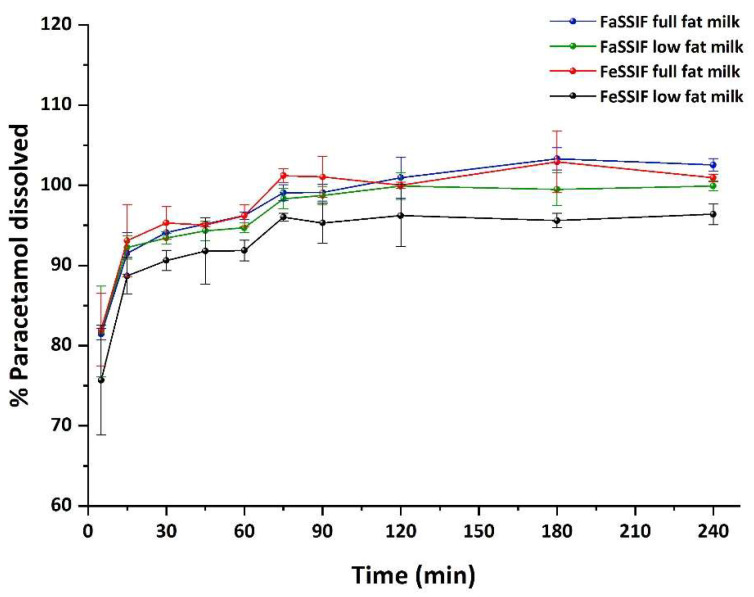
Dissolution profile of paracetamol-loaded cereal following mixing with full-fat (3.5%, blue and red lines) or low-fat (1.5%, green and black lines) milk under fasted-state gastric conditions (FaSSGF) followed by fasted-state (FaSSIF) or fed-state (FeSSIF) intestinal conditions at 37 °C (*n* = 3, ±S.D.). The red vertical line shows the time-point of medium change (60 min) (adapted from [7]).

For the simulation of the in vivo conditions of dosage form transit through the gastrointestinal tract, a two-stage dissolution study of the drug-loaded cereal was conducted in fed-state and fasted intestinal conditions. The analytical methodology was used for the quantification of PAR in these studies. Samples were taken at predetermined time intervals (0, 5, 15, 30, 60, 75, 90, 120, 180 and 240 min) and the amount of paracetamol dissolved in each medium is shown in Figure 5.

Approximately 81% of paracetamol was dissolved within the first 5 min in FaSSGF, achieving total drug dissolution in 15 min after a medium change to FeSSIF or FaSSIF. No statistically significant differences were observed in the dissolution profiles of paracetamol in the presence of either low-fat or full-fat milk and in fed-state or fasted conditions.

**Figure 6:**
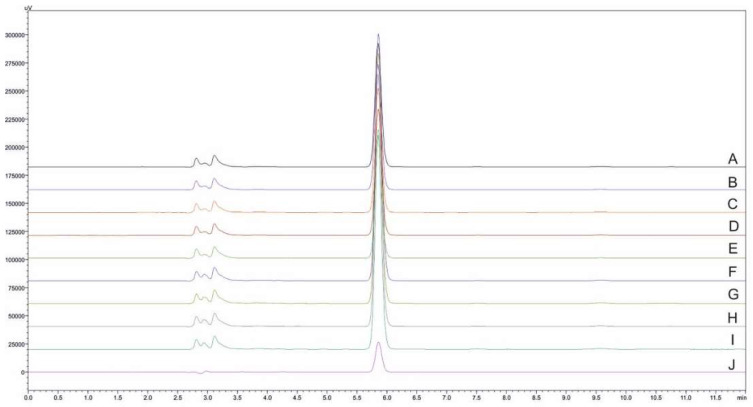

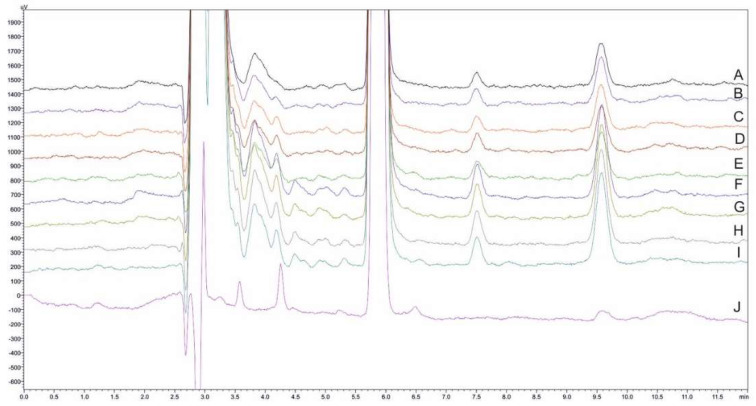
An overlay of chromatograms of the HPLC analysis of the dissolution samples taken at (**A**–**I**) 5–240 min, and (**J**) PAR standard solution (10% level). The bottom figure is a magnified overlaid chromatogram (*t*_R_, PAR = 5.8 min).

Overlaid chromatograms of the HPLC determination of PAR in a representative sample set are illustrated in Figure 6.

## 3. Materials and Methods

### 3.1. Chemicals and Solutions

The chemicals used in the present study were of at least analytical grade. Paracetamol (≥99%) was provided by Sigma-Aldrich (Steinheim, Germany). Acetonitrile, methanol (HPLC grade) and trifluoroacetic acid (≥99%) were obtained from Sigma-Aldrich (St Louis, MO, USA). The production of high-purity water was performed using a B30 purification system (Adrona SIA, Riga, Latvia). Cellulose acetate membranes (0.45 μm, Whatman^®^) were utilized for the aqueous mobile phase filtration.

Pasteurized cow milk with low fat 0.1%, medium fat 1.5% and full fat 3.6% w/w content (Declaration of Manufacturer) was purchased from local stores. These samples were stored at 4 °C until analysis.

FaSSIF, FeSSIF and FaSSGF solutions were prepared according to [7]. Briefly, an aliquot of 500 mL of FaSSGF was prepared by adding 0.9995 g of NaCl in 450 mL of H_2_O. Accordingly, the pH value of FaSSGF was adjusted to 1.6 with hydrochloric acid, and water was added to obtain a final volume of 500 mL. Then, an amount of 0.030 g of SIF Powder Original was added in 250 mL of the hydrochloric acid/NaCl solution and the volume was made up to 500 mL with the hydrochloric acid/NaCl solution.

An aliquot of 500 mL of FaSSIF double concentrate 2× was prepared by adding 0.42 g NaOH (pellets), 3.438 g NaH_2_PO_4_ and 6.186 g NaCl in 475 mL water, followed by pH adjustment to 6.8 using NaOH in order to achieve a final pH value of 6.5 after (1:1) mixing of FaSSGF: 2 × FaSSIF media. The final volume of this solution was made up to 500 mL. Accordingly, SIF Powder (2.24 g) was dissolved in 375 mL of the FaSSIF and the volume was made up to 500 mL with the same medium. The obtained medium was left to stand for 2 h prior to its use.

An aliquot of 500 mL of FeSSIF double concentrate 2× was prepared by dissolving 4.040 g of NaOH (pellets), 11.874 g of NaCl and 8.650 g of glacial CH_3_COOH in 450 mL of H_2_O, followed by pH adjustment to 5.2 in order to achieve a final pH value of 5.0 after (1:1) mixing of FaSSGF: 2 × FeSSIF media. Then, the final volume of this solution was made up to 500 mL and an amount of 11.2 g SIF Powder was dissolved in 250 mL of the FeSSIF medium, followed by volume adjustment to 500 mL with the FeSSIF. The obtained medium was left to stand for 2 h prior to its use.

### 3.2. Instrumentation and Chromatographic Conditions

All analyses were carried out on the HPLC Shimadzu system, which was composed of two LC-20AD high-pressure pumps, a CTO-20AC thermostated column compartment, an SIL-20AC HT thermostated autosampler and an SPD-20A PDA detector (Kyoto, Japan). The analytical column was a reversed-phase BDS Hypersil C_18_ (250 × 4.6 mm, 5 μm, Thermo Fisher Scientific, Waltham, MA, USA). Data acquisition and instrument control were performed using Lab Solutions software (version 5.42 SP3).

The mobile phase consisted of phosphate buffer (25 mM, pH = 3) and methanol at 80:20, v/v (isocratic elution). The flow rate was set to 1 mL min^−1^ and the chromatographic run lasted 12 min. An injection volume of 10 μL was chosen to reduce the exposure of the stationary phase to potential fat and protein residues. The column was maintained at 30 °C. The analyte was detected spectrophotometrically at its maximum wavelength of 243 nm to reduce absorbance interferences from the dissolution medium. The samples were maintained at 4 °C in the autosampler tray. Between the injection of different samples, the autosampler was sequentially washed with 500 μL of water: CH_3_OH (50:50, v/v) mixture to remove any residual sample.

### 3.3. Preparation of Calibration (CS) and Validation Standards (VS)

Aqueous stock solutions of PAR (6000 μg mL^−1^) were prepared and stored at 4 °C. Working solutions were used on the same working day. Validation standards (VS) and calibration standards (CS) were made by serial dilution of the stock solutions on three successive days (*n* = 3). Five concentration levels (*m* = 5), namely 10, 50, 100, 300 and 600 μg mL^−1^ (which corresponded to 2, 10, 20, 60 and 120% of the highest expected PAR concentrations after the complete dissolution of a 3D-printed formulation), were prepared in three replicates (*n* =3) for each experiment series (*k* = 3). Calibration standards were used to determine the most appropriate response function, allowing the quantification of samples.

Validation standards were prepared in the same way as the CS in each milk-containing biorelevant medium (FaSSGF: milk, FaSSIF: milk or FeSSIF: milk, 4:1 in all cases); the stock solutions were spiked with appropriate amounts of paracetamol to obtain the desired concentration level (2–120%). To investigate the potential effect of the fat content (drug partition to fat) on the PAR determination, full-fat milk (3.6%) was used as the worst-case scenario in terms of matrix complexity. Each concentration level was analyzed in three replicates (*n* = 3).

### 3.4. Sample Preparation

Dissolution samples were collected in 1.5 mL Eppendorf tubes and stored at −18 °C until their analysis. A volume of 500 μL of dissolution or blank sample was mixed with 50 μL of water (blank sample) or analyte working solution to obtain the desired concentration level, vortexed for 10 s and left to equilibrate for 15 min. An aliquot of 450 μL of the ice-cold precipitation reagent (acetonitrile, methanol or 10% v/v TFA aqueous solution) was then added to precipitate the proteins in the milk-containing dissolution samples. Then, the obtained mixture was subjected to vortex irradiation and centrifugation at 4000 rpm for 5 min. An aliquot of 200 μL of the clear supernatant was diluted 5-fold with water and analyzed by HPLC.

### 3.5. Method Validation

The herein developed method in dissolution samples was validated using the total error approach established by “*Société française des sciences et techniques pharmaceutiques*” (SFSTP), which is a standardization body related to the pharmaceutical industry. It is a decision-making graphical tool that helps the analyst to examine the validity of an analytical protocol. The upper and the lower *β*-ETI, the intermediate precision (s_r_ %) and the mean relative bias were evaluated through the calculation of the back-calculated concentrations that corresponded to the validation standards through the linear model. Moreover, accuracy profiles at the probability of 95% were plotted to assess its suitability. In this way, the results that might fall outside the acceptance limits *λ* are expected to be less than 5% [18,20,21,22]. In order to fulfil the conditions of a valid analytical method, the *β*-ETI must be included inside the range −*λ* and +*λ*. The mathematical expression that describes the analytical profile is the following:

$$\left[bias{\left(\%\right)}_{j}-Q_{t}\left(v;\frac{1+\beta }{2}\right)\sqrt{1+\frac{1}{pnB_{j}^{2}}s_{r,j}\;};bias{\left(\%\right)}_{j}+Q_{t}\left(v;\frac{1+\beta }{2}\right)\sqrt{1+\frac{1}{pnB_{j}^{2}}s_{r,j}\;}\right]$$

where *bias(%)_j_* = $\frac{{\hat{\mu }}_{j}-\mu_{Tj}}{\mu_{Tj}}\times 100$, s*_r,j_* = $\frac{{\hat{\sigma }}_{W,j}^{2}+{\hat{\sigma }}_{B,j}^{2}}{{\hat{\mu }}_{j}}\times 100$, *B_j_* =$\sqrt{\frac{\frac{{\hat{\sigma }}_{Β,j}^{2}}{{\hat{\sigma }}_{W,j}^{2}}+1}{n\frac{{\hat{\sigma }}_{Β,j}^{2}}{{\hat{\sigma }}_{W,j}^{2}}+1}}$, ν = $\frac{{\left(R+1\right)}^{2}}{R+\frac{1}{n\left(p-1\right)}+1-\frac{1}{pn^{2}}}$,

- ${\hat{\mu }}_{j}$ is the estimate of the mean results of the *j^th^* concentration level;
- ${\hat{\mu }}_{T}$ is the unknown “true value”;
- *p* is the number of series;
- *n* is the number of independent replicates per series;
- *Q_t_* (*ν*;$\;\frac{1+\beta }{2})$ is the *β* quantile of the *t*-Student distribution with *ν* degrees of freedom;
- ${\hat{\sigma }}_{W,j}^{2}$ is the within-series variance;
- ${\hat{\sigma }}_{B,j}^{2}$ is the between-series variance.

### 3.6. System Suitability Test

A system suitability test (SST) was established to examine the system’s performance for the intended application. On this basis, six replicates of 10 μg mL^−1^ were performed to determine the column efficiency, *N* (in terms of number of theoretical plates), retention time, injection repeatability and tailing factor (*T*_f_). The acceptance criteria were considered as *N* > 5000, RSD ≤ 2% for both retention time and peak area and *T*_f_ not more than 1.5.

### 3.7. Application of the Proposed Method—Dissolution Test

Two-stage dissolution studies were conducted in 100 mL FaSSGF pH 1.6 (6 g cereal that corresponded to 50 mg paracetamol in 25 mL milk and 75 mL FaSSGF) for 1 h. Accordingly, the studies were performed in FeSSIF pH 5.0 or FaSSIF pH 6.5 (final volume: 200 mL), for 3 h at 37 °C and at a paddle rotational speed of 50 rpm (PT DT7 USP Apparatus II Dissolution Test Instrument, Pharma Test Ltd., Hainburg, Germany). Two-milliliter samples were withdrawn and replaced at predetermined time points. For the 60-min sample, the addition of 100 mL of pre-heated 2 × FaSSIF pH 6.8 or 2 × FeSSIF pH 5.2 took place in order to obtain a medium with a total volume of 200 mL and a pH value of ca. 6.5 or 5.0, respectively. Samples were withdrawn (2 mL) from the mixed media and were replaced with FaSSGF + 2 × FaSSIF 1:1 or FaSSGF + 2 × FeSSIF 1:1 preheated at 37 °C.

## 4. Conclusions

In this work, an isocratic HPLC method was developed for the determination of paracetamol in milk-based biorelevant dissolution media obtained from a 3D-printed formulation for pediatric use. The milk-based biorelevant dissolution medium is considered representative of the fasted and fed state. Under the optimized conditions, high recovery values of paracetamol were obtained in all biorelevant media tested. The method is characterized by simplicity and cost-effectiveness. Moreover, it results in high throughput, making it useful for the routine analysis of these biorelevant media for the investigation of dissolution tests of paracetamol-containing dosage forms. The development of such methodologies is possible to encourage the analysis of solid dosage forms for in vitro testing in complex media. This kind of test aims to improve the understanding of in vivo behavior, and they can potentially assist towards the development of delivery systems characterized by high robustness.

## Figures and Tables

**Table 1:** Comparison of precipitation reagents (MeOH, ACN, 10% v/v TFA) on the matrix effect.

Calibration Curve ^1^	Slope ± SD ^2^ (×10^3^)					
Aqueous	21.1 ± 0.10					
**Precipitation reagent/fat content**	**MeOH**		**ACN**		**10% TFA**	
	Slope ± SD (×10^3^)	ME ^3^ (%)	Slope ± SD (×10^3^)	ME%	Slope ± SD (×10^3^)	ME%
[FaSSGF:milk, 4:1]						
0.1%	20.7 ± 0.31	98.1	21.4 ± 0.26	101.4	20.8 ± 0.31	98.6
1.5%	20.7 ± 0.10	98.1	20.9 ± 0.07	99.1	20.4 ± 0.07	96.7
3.6%	21.0 ± 0.08	99.5	22.0 ± 0.28	104.2	21.4 ± 0.16	101.4
[FaSSIF:milk, 4:1]						
0.1%	19.4 ± 0.28	91.9	20.8 ± 0.36	98.6	20.2 ± 0.17	95.7
1.5%	20.3 ± 0.06	96.2	19.0 ± 0.22	90.0	19.6 ± 0.22	92.9
3.6%	20.9 ± 0.17	99.1	21.5 ± 0.12	101.9	19.7 ± 0.14	93.4
[FeSSIF:milk, 4:1]						
0.1%	19.5 ± 0.31	92.4	20.8 ± 0.45	98.6	20.1 ± 0.26	95.3
1.5%	20.6 ± 0.28	97.6	19.7 ± 0.17	93.3	19.2 ± 0.43	91.0
3.6%	20.5 ± 0.23	97.2	21.3 ± 0.20	101.0	20.1 ± 0.35	95.3

^1^ Constructed using five calibration levels of 2–120%. ^2^ Standard deviation of the slopes. ^3^ Matrix effect was calculated as ratio of the slopes of the matrix-matched (individual sample) to the aqueous calibration curves.

**Table 2:** Validation results for the determination of PAR in milk-containing biorelevant dissolution samples.

Validation Criteria			
Response function (linear regression)	Slope (×10^3^)	Intercept (×10^3^)	*r* ^2^
(*k* ^1^ = 3; *m* = 5; *n* = 3) (2–120%)			
Day 1	21.07	−2.483	0.9997
Day 2	21.13	−0.238	0.9999
Day 3	21.18	−3.135	1.0000
Precision (*k* = 3; *n* = 3)			
C (μg mL^−1^)	s_r_ (%) ^2^	s_R_ (%) ^3^	
10	2.1	2.6	
50	2.7	3.0	
100	1.9	3.1	
300	1.5	2.4	
600	1.4	2.5	
Trueness (*k* = 3; *n* = 3)			
C (μg mL^−1^)	Relative bias (%)		
10	+1.1		
50	+3.9		
100	−1.2		
300	−3.0		
600	−4.5		
Accuracy (*k* = 3; *n* = 3)			
C (μg mL^−1^)	Relative *β*-ΕΤΙ (%)		
10	[−6.13, 8.25]		
50	[−11.67, 3.81]		
100	[−11.75, 9.28]		
300	[−11.00, 5.10]		
600	[−13.34, 4.29]		
Linearity (*k* = 3; *n* = 3; *m* = 5) (2–120%)			
Slope	1.000		
Intercept	−0.0004		
*r* ^2^	0.9999		
LOD (μg mL^−1^)	0.02		
LLOQ/ULOQ (%)	2/120		

^1^*k*, *m* and *n* correspond to the number of experiments, calibration levels and replicates, respectively. ^2^ s_r_ (%): relative standard deviation under repeatability conditions. ^3^ s_R_ (%): relative standard deviation under intermediate precision.

## Data Availability

Data is contained within the article.
